# Cerebral Small Vessel Disease Burden Related to Carotid Intraplaque Hemorrhage Serves as an Imaging Marker for Clinical Symptoms in Carotid Stenosis

**DOI:** 10.3389/fneur.2021.731237

**Published:** 2021-10-14

**Authors:** Xiaoyuan Fan, Xiaoqian Zhang, Zhichao Lai, Tianye Lin, Hui You, Changwei Liu, Feng Feng

**Affiliations:** ^1^Department of Radiology, Peking Union Medical College Hospital, Chinese Academy of Medical Sciences and Peking Union Medical College, Beijing, China; ^2^Department of Vascular Surgery, Peking Union Medical College Hospital, Chinese Academy of Medical Sciences and Peking Union Medical College, Beijing, China; ^3^State Key Laboratory of Difficult, Severe and Rare Diseases, Peking Union Medical College Hospital, Chinese Academy of Medical Sciences and Peking Union Medical College, Beijing, China

**Keywords:** cerebral small vessel disease, carotid stenosis, carotid intraplaque hemorrhage, magnetic resonance imaging, stroke

## Abstract

**Objectives:** In patients with carotid stenosis, to investigate the relationship between carotid intraplaque hemorrhage (IPH) and total burden of cerebral small vessel disease (CSVD) and preliminarily explore whether the total CSVD burden as an imaging marker can distinguish the severity of clinical symptoms.

**Methods:** A total of 108 patients (the mean age was 66 ± 7 years, and 85.2% were male) with unilateral carotid stenosis ≥50% underwent brain MRI and high-resolution MRI for carotid plaque characterization. The total burden of CSVD was calculated by accumulating one point according to the presence or severity of each of the four MRI markers: white matter hyperintensities, lacunes, perivascular spaces, and cerebral microbleeds. Recent clinical symptoms including transient ischemic attack, amaurosis fugax, and ischemic stroke were recorded. The association between intraplaque hemorrhage (IPH) and total CSVD burden was examined adjusted for other risk factors. The symmetry of CSVD burdens between the ipsilateral and contralateral hemispheres of IPH was tested. Imaging features (CSVD score, IPH, degree of stenosis, and completeness of the circle of Willis) were correlated with clinical symptoms by Kruskal–Wallis H test, Chi-square test, and Fisher's exact test.

**Results:** Multivariable logistic regression analysis showed that IPH (OR = 2.98, 95% CI [1.39, 6.40], *p* = 0.005) was independently associated with a higher CSVD score. The presence of unilateral IPH was associated with the inter-hemispheric CSVD score difference (*p* = 0.004). Patients with stroke had a higher ipsilateral CSVD score than asymptomatic patients (*p* = 0.004) and those with transient ischemic attack/amaurosis fugax (*p* = 0.008). The statistical difference was marginally significant between symptoms and IPH (*p* = 0.057). No statistical difference was found between the symptoms and degree of stenosis and the completeness of the circle of Willis (*p* > 0.05).

**Conclusions:** Carotid IPH is associated with an elevated total burden of CSVD in patients with carotid stenosis. Compared with the degree of stenosis, primary collaterals, and IPH, the total CSVD score might be a more effective imaging marker linked with clinical symptoms.

## Introduction

Cerebral small vessel disease (CSVD) often coexists with large artery atherosclerosis (LAA) (1, 2). CSVD can be observed in about two-thirds of patients with carotid stenosis (3). In patients with carotid stenosis, CSVD is associated with increased risks of long-term cardiovascular death (3) and future stroke (4, 5) and adversely affects postoperative cognition (6). Therefore, a better understanding of the pathogenesis for CSVD in carotid stenosis would help improve patients' prognosis.

MRI-defined carotid intraplaque hemorrhage (IPH) is in strong agreement with the histologically defined vulnerable plaque (7) and considered as a reliable marker of thromboembolic plaque activity (8). By releasing emboli into the cerebral vascular system, IPH may be an important risk factor for CSVD. Previous researches (9–13) and meta-analyses (14, 15) reached controversial conclusions on the association between plaque characteristics and CSVD. Most previous studies (9–13) did not provide an adequate control of confounders, possibly interrelated and patient-related. Moreover, previous studies (9–13) often focused on an isolated CSVD marker. Actually, multiple CSVD imaging features are often present simultaneously or sequentially; thus, CSVD should be treated as a whole brain disease (16). A prevailing scoring system called the “total burden of CSVD” expressed by the cooccurrence of white matter hyperintensities (WMHs), lacunes, perivascular spaces (PVSs), and cerebral microbleeds (CMBs) has been proposed for capturing the overall brain damage resulting from CSVD, rather than estimating only one or two individual CSVD markers separately (17). However, the association between carotid IPH and total CSVD score has not been confirmed in carotid stenosis patients.

In addition, although IPH is a strong predictor of stroke in carotid stenosis patients (18), carotid plaque MRI examination is not often performed in clinical practice. If the total CSVD burden is associated with presence of IPH, it may be another effective imaging marker for indicating clinical symptoms. Actually, previous longitudinal studies (4, 5) demonstrated that WMHs and lacunes can identify a high-risk group for ischemic events in patients with carotid stenosis. Similarly, whether “the total CSVD burden” can distinguish the severity of symptoms in carotid stenosis has not been reported.

Therefore, the main purpose of our study was to investigate the relationship between carotid IPH and the total burden of CSVD by adding a within-patient design. The additional purpose was to preliminarily test whether the total CSVD score was an effective imaging marker linked with clinical symptoms.

## Methods and Materials

### Participants

**Figure 1:**
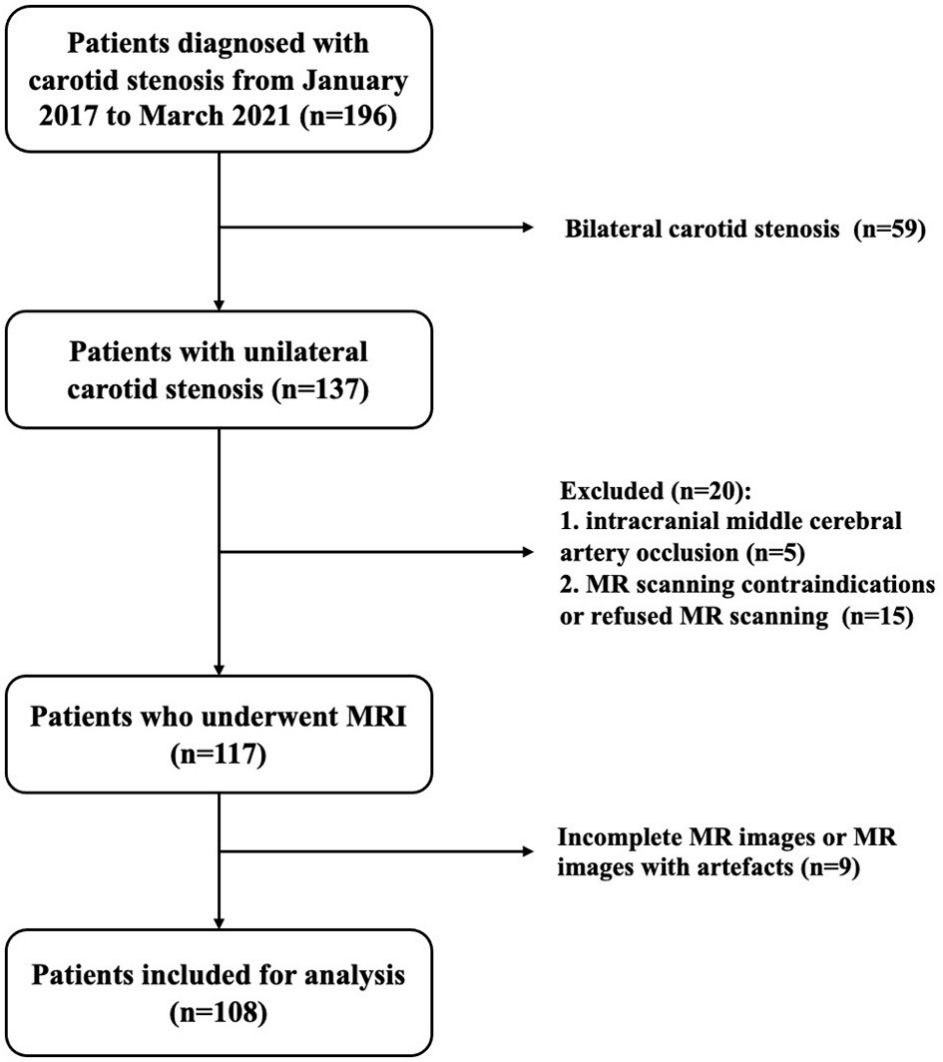
Flowchart of patient enrollment.

This study was approved by the Medical Ethics Committee of the Peking Union Medical College Hospital, in line with the Declaration of Helsinki. All participants provided written informed consent for this study. Patients were recruited from a cohort study in vascular surgery, designed to explore the application value of multimodal MRI in carotid stenosis patients (19). Patients with unilateral carotid stenosis ≥50% diagnosed with computed tomography angiography (CTA) were included. The exclusion criteria included: (a) patients with intracranial large artery stenosis (≥50%) or occlusion; (b) patients with MR scanning contraindications or who refused MR scanning; and (c) incomplete MR images or MR images with artifacts. From January 2017 to March 2021, a total of 196 patients were diagnosed with carotid stenosis by CTA, and 137 patients had unilateral carotid stenosis. There were 5 patients who had intracranial middle cerebral artery occlusion, 15 patients who had MR scanning contraindications or refused MR scanning, and 9 patients who met incomplete MR images or MR images with artifacts to be excluded. Finally, 108 patients were included in our study (Figure 1).

Data on systemic vascular risk factors were obtained from medical records. Clinical symptoms were recorded by a vascular surgeon with more than 10 years of experience. Symptomatic carotid stenosis was defined as the presence of recent (<6 months since onset) ipsilateral anterior circulation transient ischemic attack (TIA), amaurosis fugax, and non-disabling ischemic stroke (20).

### Magnetic Resonance Imaging

All MR examinations were performed on a 3.0 T scanner (GE Discovery 750). Brain MRI was performed with an 8-channel phased-array head coil, and carotid plaque MRI was performed with 8-channel or 32-channel phased-array head and neck coil. High-resolution MRI for carotid plaque characterization was performed with 3D fat-suppressed fast-spin-echo T1-weighted black blood sequence: slice thickness = 0.8 mm, time of repetition = 800 ms, echo time = 15.7 ms, flip angle = 90°, field of view = 204 × 184 mm^2^, and acquisition matrix = 320 × 256. The parameters of conventional MRI sequences are shown in Supplementary Table 1.

### Evaluation of CSVD and IPH

All four MRI markers of CSVD (lacunes, WMHs, PVSs, and CMBs) were identified according to the previously reported neuroimaging standards (21). The detailed evaluation criteria of each CSVD marker and the total CSVD burden score are shown in Supplementary Table 2. Briefly, the deep white matter and periventricular white matter were graded separately according to the Fazekas scale (22). We graded the number of PVSs in the basal ganglia with a three-category ordinal scale as follows: 0–10 (category 1); 11–25 (category 2); >25 (category 3) (23). The number of lacunes and CMBs was also recorded. CMBs strictly located in lobes were not recorded. For each patient, an overall CSVD burden score was calculated by counting one point according to the presence of four CSVD markers (17). The overall CSVD score ranged from 0 to 4. The CSVD scores in ipsilateral and contralateral hemispheres were scored separately.

IPH was identified according to the presence of a hyperintense region in the atherosclerotic plaque on the high-resolution MRI for carotid plaque characterization. The presence of hyperintensity was defined on the basis of a signal intensity ≥150% of that of the adjacent skeletal muscle (24).

**Figure 2:**
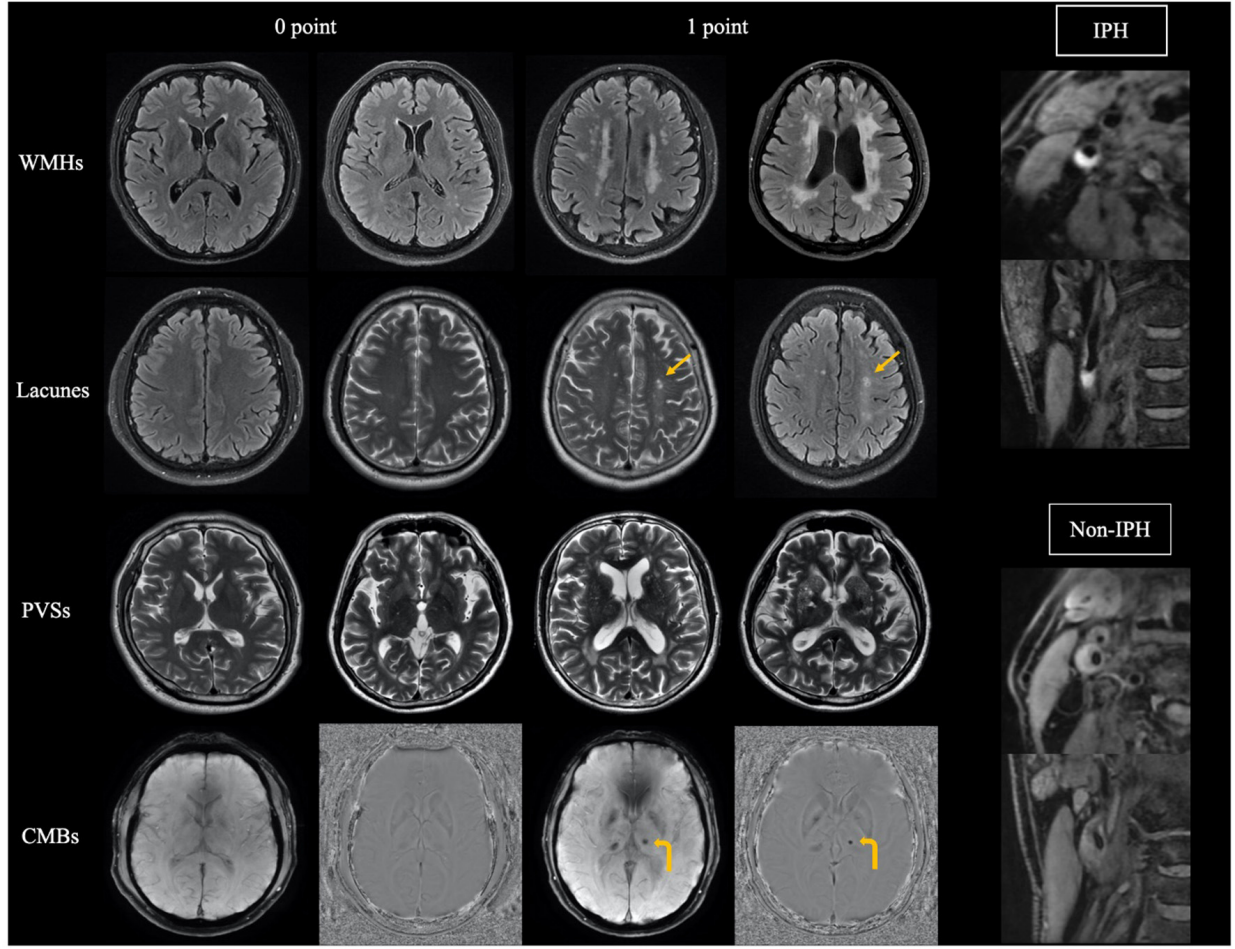
Reference images of total cerebral small vessel disease burden and carotid intraplaque hemorrhage (IPH). Arrow indicates lacunes, and curved arrow indicates cerebral microbleeds. WMHs, white matter hyperintensities; PVSs, perivascular spaces; CMBs, cerebral microbleeds.

Reference images of the total CSVD burden and IPH are shown in Figure 2. Two radiologists (H. You and T. Lin, with 19 years and 7 years of neuroradiology experience, respectively) evaluated the CSVD score and the presence of IPH. They were trained before evaluation and viewed the MRI images blinded for the clinical information. Disagreements were resolved by consensus.

### Other Imaging Features

The degree of stenosis was measured with CTA. Moderate or severe stenosis was determined according to the North American Symptomatic Carotid Endarterectomy Trial criteria (NASCET) grading (25), and we also carefully performed the diagnosis of carotid near-occlusion based on previous studies (26).

The completeness of the circle of Willis was evaluated based on the presence of anterior communicating artery and the first segments of bilateral anterior cerebral arteries (A1) on CTA. A complete circle of Willis was defined if both anterior communicating artery and bilateral A1 were visualized. An incomplete circle of Willis was defined if any of the three arterial segments was absent or hypoplastic (<0.8 mm in diameter) (27). Since the presence of posterior communicating artery had little effect on cerebral hemodynamics and clinical symptoms (28), posterior circulation arterial segments were not included.

### Statistical Analysis

The data were analyzed using IBM SPSS 25.0. Cohen's κ-value was calculated for the inter-observer agreement on the presence of IPH. Linear-weighted κ-value was calculated for the inter-observer agreement on the CSVD score. The κ ≤ 0.40 represented poor agreement, values >0.40 and ≤0.65 represented general agreement, values >0.65 and ≤0.75 represented good agreement, and values >0.75 represented excellent agreement.

To investigate the association between IPH and CSVD adjusted for other factors, logistic regression models were used for the univariable and multivariable analyses. IPH was entered as the predictor variable, and CSVD score was entered as dependent variable. We entered baseline characteristics into multivariable models according to the *p* < 0.15 in univariable model. The results are reported as odds ratios (ORs) with 95% confidence intervals (CIs). The paired Wilcoxon signed-rank test was used to test the symmetry of CSVD burdens between bilateral cerebral hemispheres. In patients with unilateral IPH, CSVD scores between ipsilateral and contralateral hemispheres of IPH were compared, while in patients with bilateral or non-IPH, the CSVD scores between ipsilateral and contralateral hemispheres of stenosis were compared. Finally, imaging markers, including ipsilateral CSVD score, IPH, the degree of stenosis, and the completeness of the circle of Willis, were compared between groups with different clinical symptoms by using Kruskal–Wallis *H*-test, Chi-square test, and Fisher's exact test adjusted by the Bonferroni correction. All *p*-values were calculated using two-tailed tests, and *p* < 0.05 was considered as statistically significant.

## Results

### Characteristics of Patients

The clinical characteristics and imaging features of patients are shown in Table 1. The mean age of patients was 66 ± 7 years, and 85.2% were male. Of the 108 patients, 66 (61.1%) had severe carotid stenosis, 26 (24.1%) had carotid near-occlusion, and 38 (35.2%) were observed with IPH. In terms of ipsilateral CSVD score, there were 41 (38%) cases of 0 point, 23 (21.3%) cases of 1 point, 23 (21.3%) cases of 2 points, 14 (13%) cases of 3 points, and 7 (6.5%) cases of 4 points. Sixty-eight (63%) patients were asymptomatic, 21 (19.4%) patients presented with TIA or amaurosis fugax, and 19 (17.6%) patients presented with stroke. Moreover, the degree of stenosis was not statistically different between patients with and without IPH (*p* = 0.293).

**Table 1:** Characteristics of the study population.

	**Total (*n* = 108)**
**Clinical characteristics**	
Age, years, mean (SD)	66.3 (7.2)
Male, *n* (%)	92 (85.2)
Hypertension, *n* (%)	73 (67.6)
Diabetes mellitus, *n* (%)	46 (42.6)
Hyperlipidemia, *n* (%)	47 (43.5)
Coronary artery disease, *n* (%)	31 (28.7)
Smoking, *n* (%)	58 (53.7)
**Imaging characteristics**	
Ipsilateral intraplaque hemorrhage, *n* (%)	38 (35.2)
Unilateral intraplaque hemorrhage, *n* (%)	31 (28.7)
*Degree of stenosis*	
Moderate stenosis, *n* (%)	16 (14.8)
Severe stenosis, *n* (%)	66 (61.1)
Near-occlusion, *n* (%)	26 (24.1)
Complete circle of Willis, *n* (%)	39 (36.1)
*Ipsilateral CSVD score*	
0, *n* (%)	41 (38)
1 point, *n* (%)	23 (21.3)
2 points, *n* (%)	23 (21.3)
3 points, *n* (%)	14 (13)
4 points, *n* (%)	7 (6.5)
*Contralateral CSVD score*	
0, *n* (%)	39 (36.1)
1 point, *n* (%)	31 (28.7)
2 points, *n* (%)	23 (21.3)
3 points, *n* (%)	7 (6.5)
4 points, *n* (%)	8 (7.4)
**Clinical symptoms**	
Absent/non-specific symptoms, *n* (%)	68 (63)
TIA/ amaurosis fugax, *n* (%)	21 (19.4)
Stroke, *n* (%)	19 (17.6)

*CSVD, cerebral small vessel disease; TIA, transient ischemic attack; n, number; SD, standard deviation*.

The inter-observer agreements on the presence of IPH and CSVD score were excellent and good with κ values of 0.84 and 0.71, respectively.

### Association Between IPH and Total CSVD Burdens

The results of univariable and multivariable logistic regression analyses for independent risk factors of ipsilateral CSVD burdens are shown in Table 2. Age, sex, hypertension, coronary artery disease, and IPH were entered into the multivariable logistic regression model. After adjustment for other risk factors, IPH (OR = 2.98, 95% CI [1.39, 6.40], *p* = 0.005) was still associated with higher CSVD burdens. There was no significant difference in the degree of stenosis (severe stenosis: *p* = 0.785; carotid near-occlusion: *p* = 0.651), completeness of the circle of Willis (*p* = 0.306), and other clinical characteristics.

**Table 2:** Multivariable logistic regression analysis for factors associated with cerebral small vessel disease burden.

	**Univariable model**		**Multivariate model**	
	**OR (95% CI)**	***P*-value**	**OR (95% CI)**	***P*-value**
Age, years	1.07 (1.02, 1.12)	0.009	1.06 (1.01, 1.12)	**0.020**
**Gender**				
Male	2.14 (0.78, 5.84)	0.138	1.49 (0.53, 4.22)	0.450
Female	1^a^	—	1^a^	—
Hypertension	1.81 (0.86, 3.79)	0.118	1.71 (0.76, 3.82)	0.194
Diabetes mellitus	0.93 (0.47, 1.86)	0.840	—	—
Hyperlipidemia	0.69 (0.35, 1.37)	0.289	—	—
Coronary artery disease	2.18 (1.03, 4.60)	0.042	1.67 (0.75, 3.72)	0.209
Smoking	1.57 (0.79, 3.13)	0.197	—	—
Ipsilateral intraplaque hemorrhage	3.59 (1.72, 7.52)	0.001	2.98 (1.39, 6.40)	**0.005**
**Degree of stenosis**				
Near-occlusion	0.77 (0.25, 2.39)	0.651	—	—
Severe stenosis	1.15 (0.43, 3.07)	0.785	—	—
Moderate stenosis	1^a^	—	—	—
Complete circle of Willis	1.45 (0.71, 2.94)	0.306	—	—

*OR, odds ratio; CI, confidence interval*.

a *Used as reference category*.

*The bold font indicates that the difference achieved statistical significance*.

**Figure 3:**
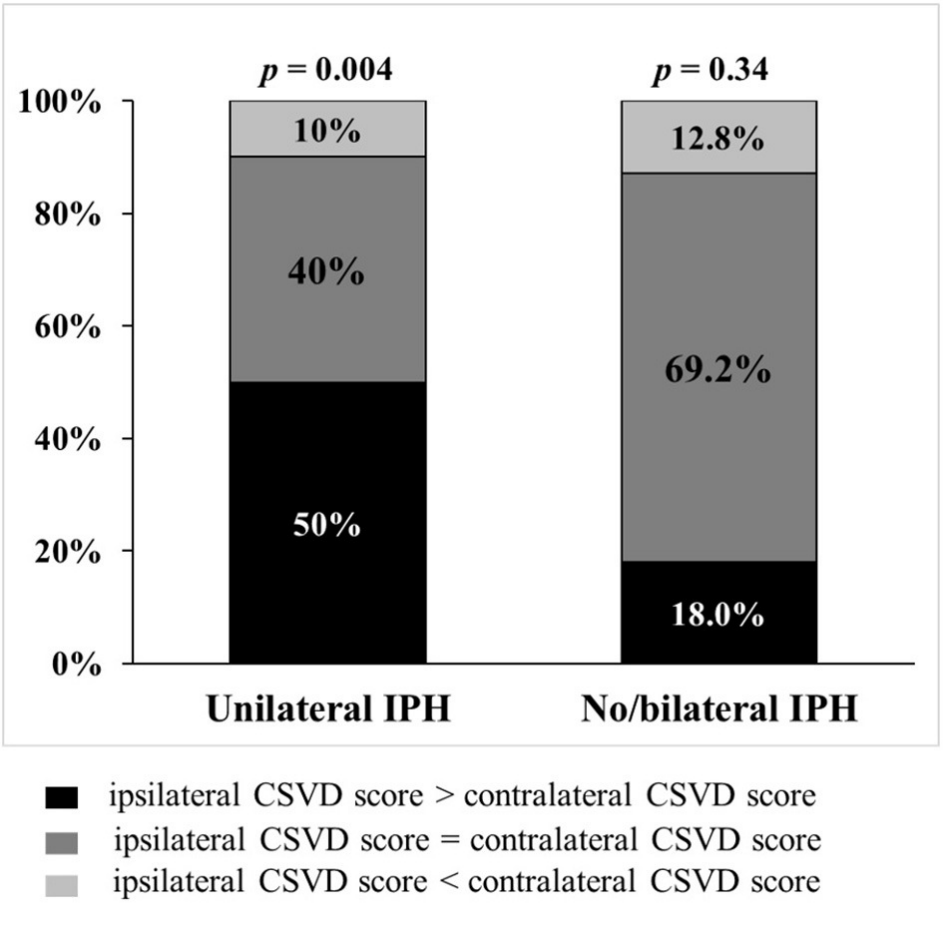
The symmetry of CSVD burden in patients with and without unilateral IPH. In 31 patients with unilateral intraplaque hemorrhage (IPH), the total cerebral small vessel disease (CSVD) scores were higher on the IPH-side (*p* = 0.004). In 77 patients with bilateral or non-IPH, CSVD scores were largely similar between ipsilateral and contralateral hemispheres of stenosis (*p* = 0.34).

**Figure 4:**
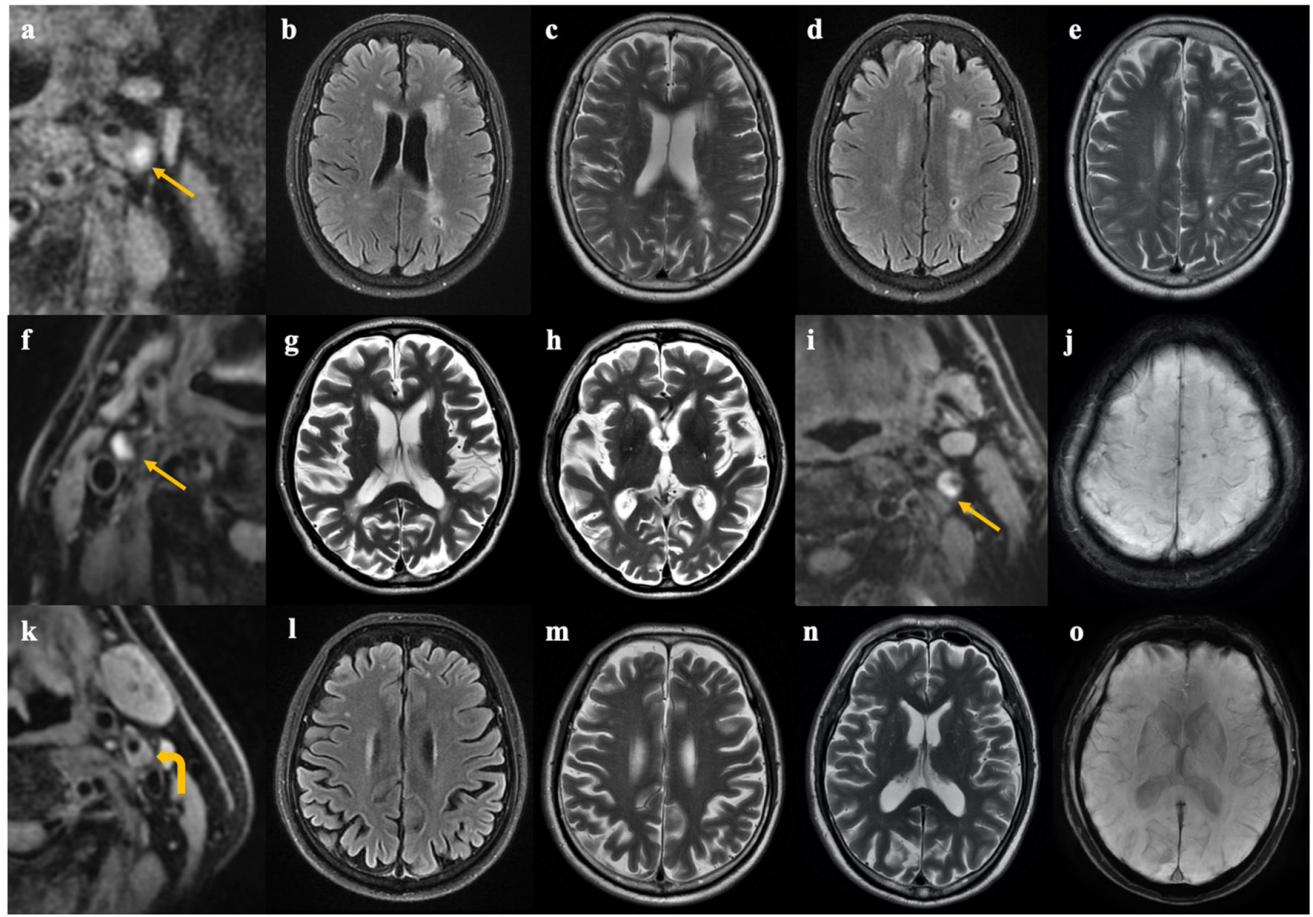
Representative cases of patients with and without unilateral carotid IPH. Case 1 **(a–e)**. A 68-year-old female complained of weakness of right limbs, and high-resolution MRI for carotid plaque showed carotid intraplaque hemorrhage (IPH) on the left side **(a)**. Brain MRI showed more severe white matter hyperintensities and lacunes on the left hemisphere than that on the contralateral hemisphere **(b–e)**. Case 2 **(f–h)**. An 82-year-old male complained of transient weakness of lower limbs. High-resolution MRI for carotid plaque showed IPH on the right side **(f)**. Brain MRI showed category-1 perivascular spaces (PVSs) in the left basal ganglia and category-2 PVSs in the right basal ganglia **(g,h)**. Case 3 **(i,j)**. An 85-year-old male complained of dizziness for 1 month. High-resolution MRI for carotid plaque showed IPH on the left side **(i)**, and a cerebral microbleed lesion was found on the ipsilateral hemisphere **(j)**. Case 4 **(k–o)**. A 71-year-old male with asymptomatic carotid stenosis. Carotid plaque without hemorrhage on the left side was shown on carotid MRI **(k)**, and cerebral small vessel disease was symmetrical between two hemispheres **(l–o)**. Arrow indicates carotid IPH, and curved arrow indicates carotid non-IPH.

IPH was associated with the asymmetry of CSVD burden between ipsilateral and contralateral hemispheres (Figure 3): In patients with unilateral carotid IPH, the CSVD scores were higher on the IPH-side (*p* = 0.004). In contrast, the CSVD scores were largely similar between ipsilateral and contralateral hemispheres of stenosis in patients with bilateral presence or absence of IPH (*p* = 0.34). Representative examples are shown in Figure 4.

### Comparison of Imaging Markers Between Groups With Different Clinical Symptoms

The comparison of imaging markers between groups with different clinical symptoms is shown in Table 3. Patients with stroke had a higher ipsilateral CSVD score than asymptomatic patients (*p* = 0.004) and those with TIA/amaurosis fugax (*p* = 0.008). IPH was observed in more than half of the patients with stroke (11 of 19, 57.9%). The statistical difference was marginally significant between symptoms and IPH (*p* = 0.057). No statistical difference was found between the degree of stenosis, the integrity of the circle of Willis, and clinical symptoms.

**Table 3:** Comparison of imaging markers between groups with different clinical symptoms.

	**Asymptomatic**	**TIA/amaurosis fugax**	**Stroke**	***P-*value**
	**(*n* = 68)**	**(*n* = 21)**	**(*n* = 19)**	
Ipsilateral CSVD score	1 [0,2]	0 [0,2]	2 [2,3]^a^	**0.003**
Intraplaque hemorrhage, *n* (%)	22 (32.4)	5 (23.8)	11 (57.9)	0.057
Degree of stenosis				0.989
Moderate stenosis, *n* (%)	11 (16.2)	3 (14.3)	2 (10.5)	
Severe stenosis, *n* (%)	41 (60.3)	13 (61.9)	12 (63.2)	
Near-occlusion, *n* (%)	16 (23.5)	5 (23.8)	5 (26.3)	
Complete circle of Willis, *n* (%)	26 (38.2)	7 (33.3)	6 (31.6)	0.830

*TIA, transient ischemic attack; CSVD, cerebral small vessel disease*.

a *Patients with stroke had higher ipsilateral CSVD score than asymptomatic patients (adjusted p = 0.004) and those with TIA/amaurosis fugax (adjusted p = 0.008)*.

*The bold font indicates that the difference achieved statistical significance*.

## Discussion

In a group of patients with unilateral carotid stenosis, we found that a significant association existed between carotid IPH and the total burden of CSVD. Moreover, compared with the degree of stenosis, primary collaterals, and IPH, the total CSVD score was a more effective imaging marker linked with clinical symptoms.

In the present study, multiple parameters including vascular risk factors, the degree of stenosis, collaterals, and carotid IPH were included to provide a comprehensive analysis on the relationship between CSVD and carotid stenosis. Theoretically, the association between LAA and CSVD may be indirect through common vascular risk factors or causative with arterial thromboembolism and impaired hemodynamics being two possible mechanisms (29). In order to better expose the effect of LAA on CSVD, we only included patients with unilateral carotid stenosis and evaluated the relationship between IPH and inter-hemispheric CSVD differences by using a within-patient design, which allowed the control of variability among individuals that may confuse the association between carotid stenosis and CSVD. By using this method, it can be further determined that the effect of carotid IPH on CSVD was independent of vascular risk factors shared by LAA and CSVD. As a reliable marker of thromboembolic plaque activity (8), the association of IPH with CSVD means that artery-to-artery thromboembolism may be an underlying mechanism through which LAA can aggravate CSVD. A previous study (30) found that IPH was also associated with decreased lumen size, which may be another source for CSVD in theory. However, in our study, the stenosis degree was not statistically different between patients with and with IPH; thus, the effect of IPH on CSVD was not driven by the association of IPH with decreased lumen size.

Our results were consistent with some previous studies evaluating the effects of carotid IPH on certain CSVD markers such as WMHs, lacunes, and CMBs (13–15). Certainly, our study also partly conflicted some studies that did not detect the association between carotid IPH and WMHs or lacunes (9–12). For example, the study by Ammirati et al. (11) examined the association between the progression of WMHs and plaque characteristics by using carotid ultrasound in asymptomatic patients with <70% stenosis, and they reached negative results. The population-based Rotterdam Study (9) involving 951 participants (only 13.1% with lacunes) also showed no association between MRI-defined carotid IPH and lacunes on MRI. The discrepancies between previous studies (9–12) and our study may stem from the different selections of study subjects, a more accurate imaging of plaque by MRI than that by ultrasound, within-subject design, and the notion of “total burden of CSVD” used in our study.

A systematic review and meta-analysis (14) of 32 studies (*n* = 17,721) showed no association of WMHs with simple carotid stenosis. Similarly, no significant association was found between the degree of stenosis, integrity of the circle of Willis, and total CSVD burden in our study. However, our results cannot negate the effect of impaired hemodynamics on CSVD because no data on cerebral blood flow or cerebrovascular reactivity were available in the present study.

In addition, our findings suggested that compared with the integrity of the circle of Willis, the degree of stenosis, and IPH, the total CSVD score was a more effective imaging marker linked with clinical manifestation. There are two main mechanisms for the occurrence of ischemic events caused by carotid stenosis; one is the downstream hypoperfusion secondary to stenosis, and the other is artery-to-artery embolism (31). The degree of stenosis, collateral circulation, or IPH can only explain a certain mechanism, while the total CSVD score can comprehensively reflect brain microstructure damage influenced by both the two mechanisms. That can partly explain why the total CSVD score was a more efficient imaging marker for the indication of clinical symptoms. Moreover, CSVD itself may accelerate the stroke process caused by carotid stenosis. The presence of CSVD often indicates impaired cerebrovascular reserve (16). When faced with decreased cerebral perfusion pressure caused by carotid stenosis, patients with CSVD would suffer from lower cerebral blood flow due to the inadequate vasodilation of cerebral micro-vessels, which promotes the occurrence of ischemic events or stroke (32).

In patients with carotid stenosis, WMHs and lacunes were reported to be associated with the risk of ischemic stroke in longitudinal studies (4, 5). Our study preliminarily extended the notion of “total burden of CSVD” to the severity of symptoms in patients with carotid stenosis. Although the present study cannot determine the predictive value of total CSVD burden for stroke, the results showed that the CSVD score can best distinguish between patients with and without stroke. That gave a hint that the total CSVD score might be a potential imaging marker for predicting clinical symptoms, which needs to be verified by future longitudinal studies.

An innovation of the present study was that we used the notion of total CSVD burden to evaluate the association between IPH, CSVD, and ischemic events. Considering the high possibility that these CSVD markers coexist with each other, merging them into the total CSVD burden can provide a more comprehensive information about the status of CSVD (17). Moreover, the total CSVD score is reliable and easy to collect as long as the standardized definition of each feature is adopted (21). When high-resolution MRI for carotid plaque characterization is not performed in patients with carotid stenosis in clinical practice, the total CSVD score evaluated on brain MRI can help indicate the stability of the plaque.

Our study also had several limitations. First, the main limitation was the cross-sectional design, and thus the causality between CSVD and clinical symptoms cannot be determined. As we mentioned above, CSVD may participate in the stroke progress caused by carotid stenosis, or their association stems from the common pathogenesis. Second, the degree of stenosis and integrity of the circle of Willis were indirect measurements of hemodynamics. Therefore, the effect of hemodynamics on CSVD or clinical symptoms cannot be directly concluded in present study. Although cerebral blood flow and cerebrovascular reactivity are more accurate indicators for cerebral hemodynamic changes, the degree of stenosis and collaterals are the commonly used imaging markers when determining treatment strategies or intraoperative procedures (25, 33). Third, our findings were only applicable to patients with carotid stenosis ≥50%. The relative contributions of IPH and hemodynamics may differ in pre-clinical subjects with <50% stenosis.

## Conclusion

In conclusion, our study demonstrated that in patients with carotid stenosis, the total burden of CSVD was associated with IPH and can be an effective imaging marker linked with clinical symptoms. Our study adumbrates the potential value of total CSVD score for risk stratification in patients with carotid stenosis. Future longitudinal studies are needed to investigate the predictive value of the total CSVD burden in patients with carotid stenosis, by which the total CSVD burden may participate in formulating treatment strategies.

## Data Availability Statement

The data that support the findings of this study are available from the corresponding author upon reasonable request.

## Ethics Statement

The studies involving human participants were reviewed and approved by Medical Ethics Committee of the Peking Union Medical College Hospital. The patients/participants provided their written informed consent to participate in this study.

## Author Contributions

XF, XZ, and ZL designed the study. XF performed the statistical analyses. XF and XZ contributed to data preparation and drafting the original manuscript. ZL was responsible for clinical evaluation. TL and XF are responsible for MR scanning. TL and HY evaluated MR images. FF and CL managed the subject recruitment. FF modified and confirmed the final article. All authors contributed to the article and approved the submitted version.

## Funding

This work was supported in part by the National Nature Science Foundation of China grant (82071899), the Beijing NaturalScience Foundation grant (L182067), and the Fundamental Research Funds for the Central Universities (3332020009).

## Conflict of Interest

The authors declare that the research was conducted in the absence of any commercial or financial relationships that could be construed as a potential conflict of interest.

## Publisher's Note

All claims expressed in this article are solely those of the authors and do not necessarily represent those of their affiliated organizations, or those of the publisher, the editors and the reviewers. Any product that may be evaluated in this article, or claim that may be made by its manufacturer, is not guaranteed or endorsed by the publisher.
