# Supplementary material for: Cerebral Small Vessel Disease Burden Related to Carotid Intraplaque Hemorrhage Serves as an Imaging Marker for Clinical Symptoms in Carotid Stenosis
Source: Front Neurol. 2021 Oct 14;12:731237. doi: 10.3389/fneur.2021.731237 (PMC8551444; doi:10.3389/fneur.2021.731237)
Supplement: Supplementary file 1 [file Data_Sheet_1.docx]

**Table S1.** Parameters for conventional MRI sequences.

| Sequences | T1WI | T2WI | FLAIR | DWI | T2*WI |
| --- | --- | --- | --- | --- | --- |
| TR (ms) | 1775 | 5258 | 12000 | 3600 | 42 |
| TE (ms) | 21.6 | 84 | 122 | 64 | 23 |
| TI (ms) | 720 | NA | 2712 | NA | NA |
| FA (°) | 111 | 142 | 160 | 90 | 20 |
| Slice thickness (mm) | 4 | 4 | 4 | 4 | 6 |
| Number of slices | 40 | 36 | 36 | 36 | 48 |
| Acquisition matrix | 320 × 160 | 320 × 320 | 288 × 192 | 128 × 128 | 480×288 |
| FOV (mm^2^) | 240 × 168 | 220 × 220 | 220 × 176 | 220 × 220 | 240×220 |
| b_max_ (s/mm^2^) | NA | NA | NA | 1000 | NA |

T1WI, T1-weighted imaging; T2WI, T2-weighted imaging; FLAIR, fluid-attenuated inversion recovery; DWI, diffusion-weighted imaging; TR, repetition time; TE, echo time; TI, inversion time; FA, flip angle; FOV, field of view.

**Table S2.** Scoring system for total CSVD burden.

| MRI markers | Definition and grades | Number or degree | Score |
| --- | --- | --- | --- |
| WMHs | WMHs were defined as hyperintensity on T2-weighted images and fluid-attenuated inversion recovery without cavitation.  Periventricular WMHs were graded as: 0 = absent, 1 = caps or pencil-thin lining, 2 = smooth halo, or 3 = irregular periventricular WMHs extending into the deep white matter. Deep WMHs were graded as: 0 = absent, 1 = punctate foci, 2 = beginning confluent foci, or 3 = large confluent areas. | Fazekas score＜2 in the deep white matter and＜3 in the periventricular white matter | 0 |
|  |  | Fazekas score ≥ 2 in the deep white matter or ≥ 3 in the periventricular white matter | 1 |
| Lacune | Lacune was defined as a round or ovoid, subcortical, fluid-filled cavity of between 3 mm and about 15 mm in diameter located in the territory of a perforating arteriole.  Number of lacunes was conservatively counted. | 0 | 0 |
|  |  | ≥ 1 lesion | 1 |
| CMBs | CMBs were defined as a small (generally 2–5 mm in diameter) area of signal void with associated blooming seen on T2*-weighted MRI.  Number of CMBs were counted. CMBs strictly located in lobes were not recorded. | 0 | 0 |
|  |  | ≥ 1 lesion | 1 |
| PVSs | PVSs were round or ovoid, with a diameter generally smaller than 3 mm and had signal intensity similar to cerebrospinal fluid on all sequences.  The number of PVSs in the basal ganglia was graded with a three-category ordinal scale as follows: 0–10 (category 1), 11–25 (category 2), and >25 (category 3). | Category-1 PVSs | 0 |
|  |  | Category-2 or -3 PVSs | 1 |

CSVD, cerebral small vessel disease; WMHs, white matter hyperintensities; CMBs, cerebral microbleeds; PVSs, perivascular spaces.
